# Interface engineering of graphene–silicon Schottky junction solar cells with an Al_2_O_3_ interfacial layer grown by atomic layer deposition

**DOI:** 10.1039/c7ra13443f

**Published:** 2018-03-16

**Authors:** Aaesha Alnuaimi, Ibraheem Almansouri, Irfan Saadat, Ammar Nayfeh

**Affiliations:** Research and Development Centre, Dubai Electricity and Water Authority (DEWA) Dubai United Arab Emirates; Department of Electrical and Computer Engineering (ECE), Masdar Institute, Khalifa University of Science and Technology P. O. Box 54224 Abu Dhabi United Arab Emirates aar.alnuaimi@gmail.com

## Abstract

The recent progress in graphene (Gr)/silicon (Si) Schottky barrier solar cells (SBSC) has shown the potential to produce low cost and high efficiency solar cells. Among the different approaches to improve the performance of Gr/Si SBSC is engineering the interface with an interfacial layer to reduce the high recombination at the graphene (Gr)/silicon (Si) interface and facilitate the transport of photo-generated carriers. Herein, we demonstrate improved performance of Gr/Si SBSC by engineering the interface with an aluminum oxide (Al_2_O_3_) layer grown by atomic layer deposition (ALD). With the introduction of an Al_2_O_3_ interfacial layer, the Schottky barrier height is increased from 0.843 V to 0.912 V which contributed to an increase in the open circuit voltage from 0.45 V to 0.48 V. The power conversion efficiency improved from 7.2% to 8.7% with the Al_2_O_3_ interfacial layer. The stability of the Gr/Al_2_O_3_/Si devices was further investigated and the results have shown a stable performance after four weeks of operation. The findings of this work underpin the potential of using an Al_2_O_3_ interfacial layer to enhance the performance and stability of Gr/Si SBSC.

## Abstract 2

One approach to improve Gr/Si SBSC performance is engineering the interface with an interfacial layer. We demonstrate the improved performance of Gr/Si SBSC upon engineering the interface with an aluminium oxide (Al_2_O_3_) layer grown by atomic layer deposition (ALD)..

## Graphical abstract

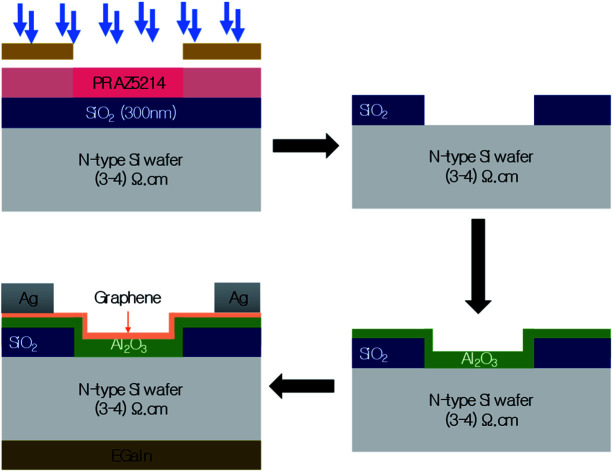

## 1. Introduction

The high cost and inadequate efficiency of existing solar cell technologies have caused current efforts to focus on investigating new materials to achieve low cost and high efficiency solar cells. Graphene is one of the most promising materials that has attracted a tremendous amount of attention in the photovoltaics field due to its unique properties, being a two-dimensional material with near-zero bandgap and highly transparent film with excellent electrical conductivity. Graphene has been utilized in various PV technologies as a transparent electrode, electron and hole transport layer in organic solar cells and catalyst in dye sensitized solar cells (DSSCs).^1–8^ The recent development of Gr/silicon (Si) Schottky barrier solar cells (SBSC) have shown a great potential to produce low cost and high efficiency solar cells with the highest reported efficiency being 15.6%.^9,10^ Despite the recent efforts in enhancing the electrical and optical performance of Gr/Si SBSC,^9–23^ further performance optimization is required. The performance of Gr/Si SBSC is highly affected by the high recombination at Gr/Si interface and the continues but non-uniform growth of native oxide that prevents the tunnelling of the photo-generated charge carriers and leading to performance degradation and instability issues. Such behaviour has been observed for metal/Si interface and among the different approaches that have been adopted to reduce the recombination and enhance the interface properties is engineering the interface with interfacial layers.^24–27^ Previous studies on Gr/Si SBSC have shown an improved performance of Gr/Si SBSC by introducing solution based interfacial layers to improve Gr/Si interface.^15,18,19,21,22^ However, some of the undesirable properties of solution processed materials are the difficulty in obtaining uniform coating and controlling the thickness precisely which are important properties to obtain good passivation at the interface that doesn't affect the tunnelling of the charge carriers. The ALD technique is an excellent choice for the deposition of high quality thin film owning to the ability of obtaining conformal coating and controlling the thickness and composition at the atomic level. Herein, we analyzed the effect of Al_2_O_3_ grown by ALD as interfacial layer for Gr/Si SBSC.

## 2. Experimental methods

### 2.1 Graphene synthesis

**Fig. 1:**
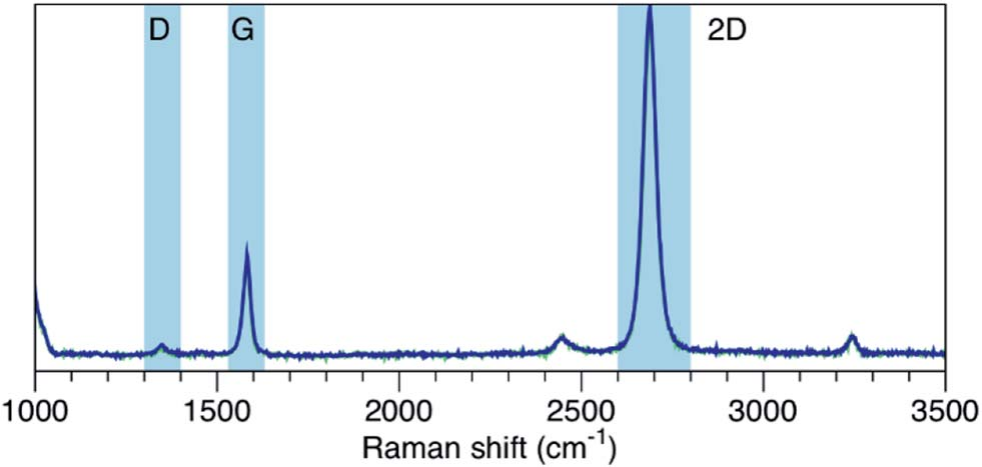
Raman spectrum of synthesized graphene.^28^

A high quality monolayer graphene with sheet resistance of ∼650 Ω sq^−1^ was grown on copper foil using chemical vapour deposition using Aixtron cold wall CVD reactor. The growth was done using a mixture of methane CH_4_ (15 sccm) and hydrogen H_2_ (60 sccm) at 1060 °C and pressure of 15 mbar. Further details about the synthesis can be found in ref. 28. The formation of high quality monolayer graphene was examined using Raman analysis. Fig. 1 shows the Raman spectrum of as-synthesized graphene. The ratio of 2D-band peak (2682 cm^−1^) to G-band-peak (1580 cm^−1^) *I*_2D_/*I*_G_ is greater than two which confirms the formation of a monolayer graphene. The weak D-band peak (1350 cm^−1^) suggests the high quality of the grown graphene.

### 2.2 Gr/Si solar cells fabrication

**Fig. 2:**
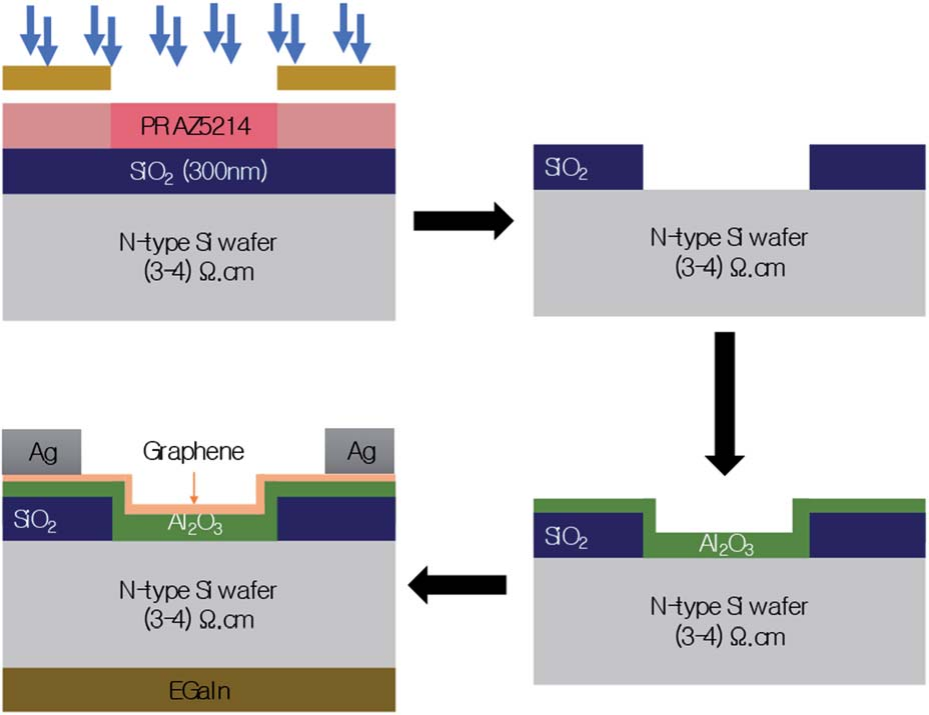
Schematic diagram of Gr/Al_2_O_3_/Si solar cell fabrication steps.

The fabrication steps of Gr/Si SBSC are illustrated in Fig. 2. Twelve solar cells with active area of 2 × 2 mm were fabricated using lightly doped n-type Si substrate with resistivity of 3–4 Ω cm. A 300 nm silicon oxide was deposited on top of the silicon substrate to define the active area. Using lithography, a window was patterned and the silicon oxide was etched using buffered oxide etch (BOE) to expose the underlying silicon which defines the size of the solar cell. After the etching step, the samples were transferred to the Oxford instruments FlexAl ALD reactor for Al_2_O_3_ deposition.

A thin layer of Al_2_O_3_ oxide was deposited using thermal ALD at low temperature of 300 °C and pressure of 200 mTorr. Trimethylaluminum (TMA, Al(CH_3_)_3_) was used as the gas precursor for Al while H_2_O is used the oxygen precursor. The estimated deposition rate is 1 Å per cycle and the estimated thickness is ∼20 Å. The ALD step was followed by graphene transfer. Two layers of graphene were transferred using the standard PMMA (poly(methyl methacrylate)) transfer process. The samples were exposed to the vapour of nitric acid for 1 min to dope the graphene. Gallium–indium eutectic (Ga–In 99.99%) was used as the back silicon contact while silver (Ag) paste was applied around the active area to contact the graphene.

### 2.3 Gr/Si solar cells characterization

Sol3A 94123A solar simulator was used to measure the current–voltage characteristics of the fabricated cells under 1 sun illumination. A black tape was used to cover the solar cell except at the active area to avoid any current collected from surrounding area. The light intensity of the simulator was calibrated using silicon reference cell and irradiance monitor. Agilent Probe analyser was used for the dark current measurements.

## 3. Results and discussion

**Fig. 3:**
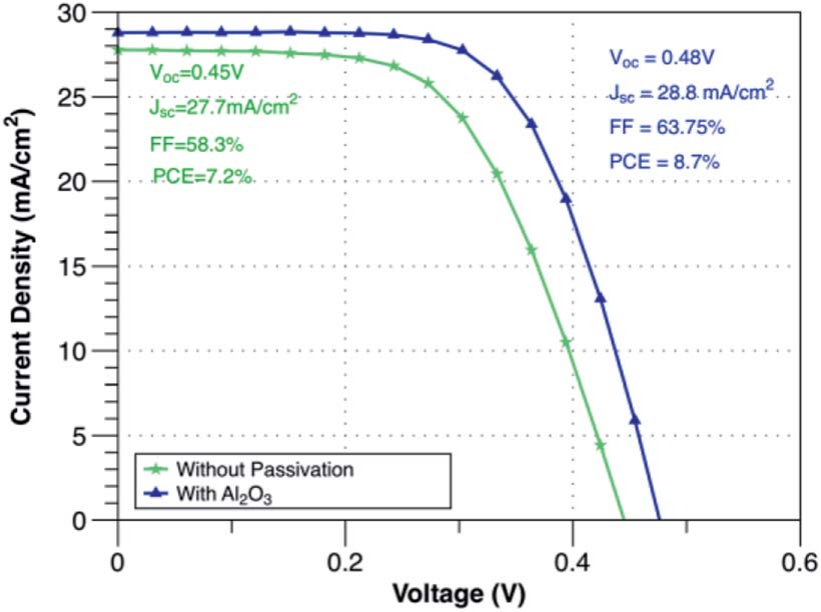
*J*–*V* characteristics of Gr/Si solar cells with and without Al_2_O_3_ interfacial layer.

**Fig. 4:**
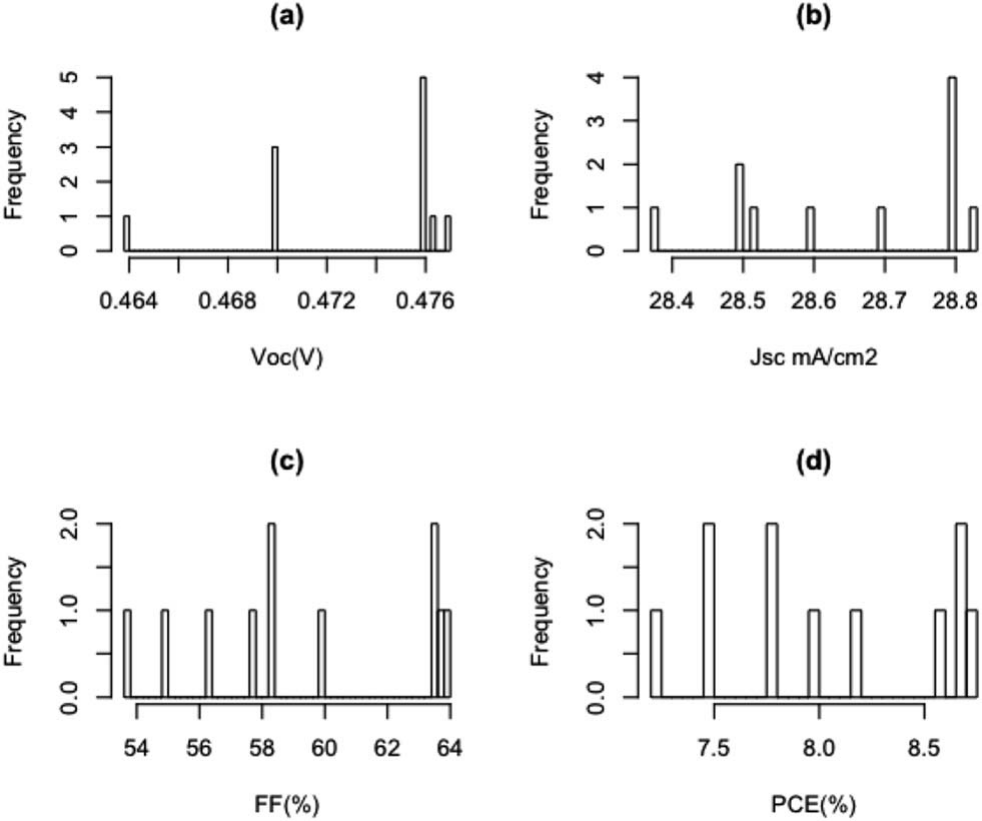
Histogram of (a) open circuit voltage (b) short circuit current (c) fill factor (d) power conversion efficiency for Gr/Si solar cells with Al_2_O_3_ interfacial layer.

Fig. 3 illustrates the current density–voltage (*J*–*V*) curves under AM1.5 illumination conditions of Gr/Si solar cells with and without Al_2_O_3_ interfacial layer. In the absence of Al_2_O_3_ interfacial layer, the short circuit current density (*J*_sc_), open circuit voltage (*V*_oc_) and fill factor (FF) of Gr/Si solar cell are 27.7 mA cm^−2^, 0.45 V, 58.3%. With the introduction of Al_2_O_3_ interfacial layer, the performance of the cell improved significantly. The *J*_sc_, *V*_oc_, and FF increased to 28.8 mA cm^−2^, 0.48 V, 63.75% leading to an enhancement in the power conversion efficiency from 7.2% to 8.7%. Table 1 shows the average value of performance parameters of devices fabricated with and without Al_2_O_3_ passivation layer. Fig. 4 shows the histogram of keys performance parameters (*V*_oc_, *J*_sc_, FF and PCE) for Gr/Si solar cells with Al_2_O_3_ interfacial layer.

**Table 1:** Summary of performance parameters of devices fabricated with and without Al_2_O_3_ passivation layer

Solar cell	*V*_oc_ (V)	*J*_sc_ mA cm^−2^	FF (%)	PCE (%)
Without passivation	0.43	27.7	53.9	6.4
With Al_2_O_3_	0.47	28.6	59.2	8.0

**Fig. 5:**
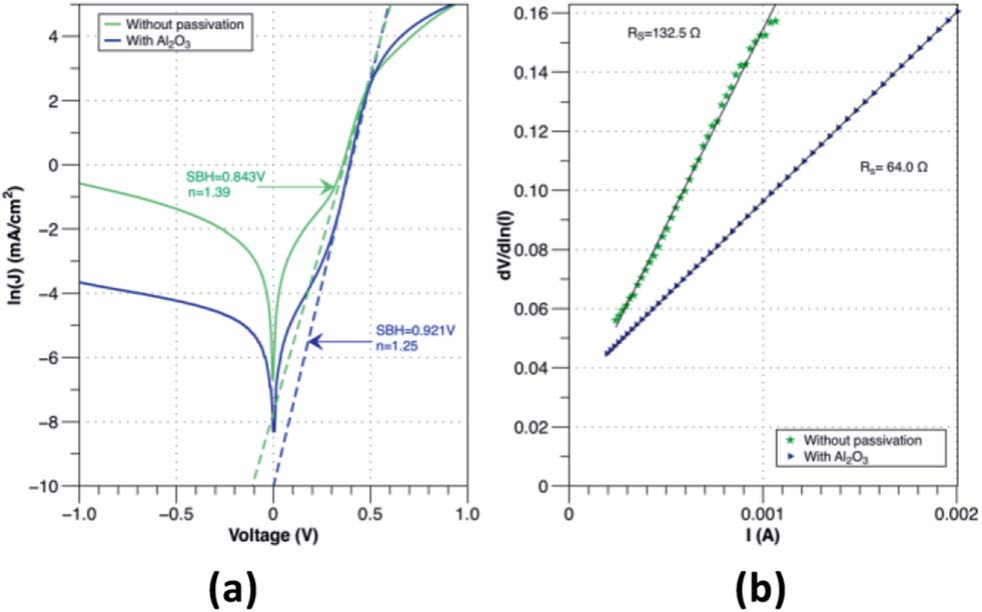
(a) Dark *J*–*V* characteristics of Gr/Si solar cells with and without Al_2_O_3_ interfacial layer. Inset shows the corresponding ln *J*–*V* curves. (b) Plots of d*V*/dln(*I*) *versus I* for Gr/Si solar cells with and without Al_2_O_3_ interfacial layer.

One of the main factors that contributed to the improvement in the solar cell performance with the addition of Al_2_O_3_ interfacial layer is the increase in the Schottky barrier height (SBH). The SBH was extracted from the slope of the dark ln(*J*)–*V* curve at the forward bias linear region as illustrated in Fig. 3a. For Gr/Si solar cells without Al_2_O_3_, the estimated SBH is 0.843 V and it increased to 0.921 V with Al_2_O_3_ interfacial layer. The increase in the barrier height at the interface blocks the transport of the photo-generated electrons in silicon hence reducing the leakage current. As illustrated in Fig. 5a, the reverse saturation current is reduced from 4.50 × 10^−4^ mA cm^−2^ to 4.48 × 10^−5^ mA cm^−2^ while the ideality factor (*n*) is reduced from 1.39 to 1.25 confirming the significant reduction in the recombination of carriers. Furthermore, the increase in the SBH also indicates the creation of larger built-in potential (*V*_bi_) across the depletion region that is beneficial for enhancing the transfer of the carriers at the interface. The *V*_bi_ of the solar cell has been extracted from the crossover between the *IV* characteristics under illumination and dark condition^29^ and it was found that *V*_bi_ increased from 0.54 V to 0.58 V with Al_2_O_3_. The strong dependence of *V*_oc_ on the SBH as described in eqn (1) as well as the reduction in the dark current and ideality factor clearly explains the increase in the *V*_oc_ with the introduction of Al_2_O_3_ interfacial layer.1
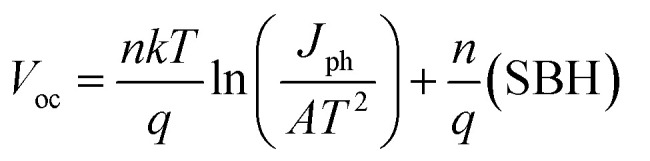


In addition, the series resistance *R*_s_ was extracted by plotting the curve of d*V*/dln(*I*) as a function of *I* and estimating the *R*_s_ value from the slope of the linear fitting to the curves to the curves (Fig. 5b). The series resistance dropped from 132.5 Ω to 64.0 Ω which results in improving the fill factor with Al_2_O_3_ interlayer.

**Fig. 6:**
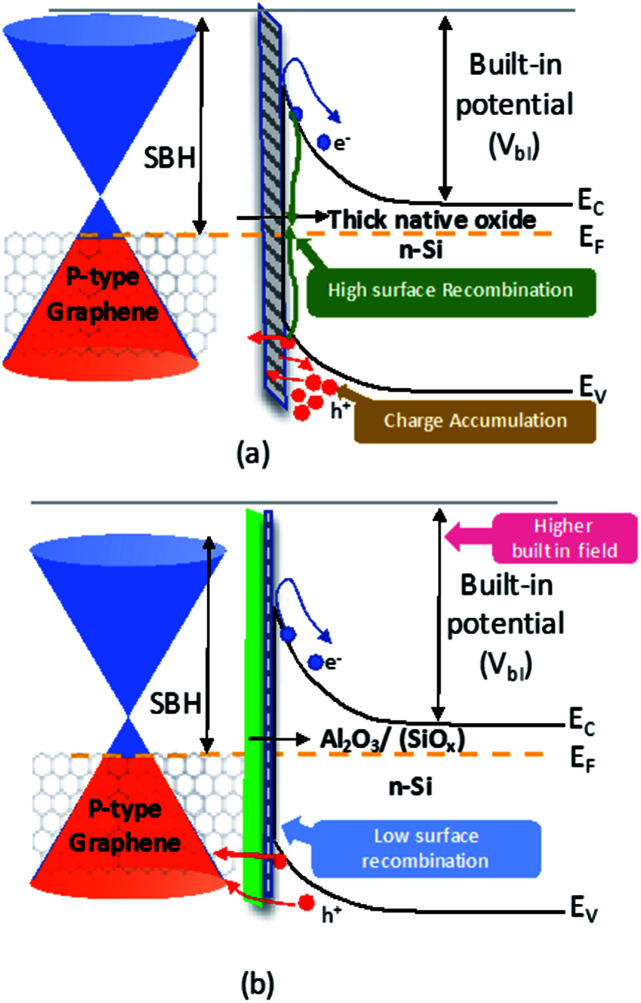
Energy band diagrams of Gr/Si SBSC (a) with thick interfacial layer (b) with Al_2_O_3_/native oxide interfacial layers.

The benefits of Al_2_O_3_ interfacial layers is not only limited to creating higher Schottky barrier but it also reduces the recombination at the interface. In SBSC the tunnelling of holes should dominates over the recombination to achieve effective charge carrier transport. According to Song *et al.*^9^ the existence of ultra-thin layer of native oxide passivates the interface between the graphene and silicon and allows the tunnelling of holes. However, since the native oxide thickness tends grow continuously, the performance of Gr/Si solar cell degrades with time. As shown in Fig. 6a, due to the increase in the native oxide thickness, the tunnelling of holes is reduced causing accumulation at the interface which consequently results in higher recombination of charge carriers. Therefore, the use of SiO_2_ interfacial layer has major issues that affect the performance stability of the Gr/Si SBSC. In contrast, Al_2_O_3_ has been proven as a good passivation layer for Si substrate.^30–33^ As shown in Fig. 6b, passivating the surface with Al_2_O_3_ immediately after the native oxide etch prevents the formation of thick native oxide at the interface. In addition, it creates a uniform and conformal Al_2_O_3_ layer that passivates the Si surface effectively. The formation of uniform ultra-thin Al_2_O_3_ layer contributed to reducing the surface recombination, improving the carrier lifetime for Si substrate and reducing the series resistance. According to Hoex *et al.*^30^ passivating the Si surface with Al_2_O_3_ reduces the density of traps significantly causing a major reduction in the surface recombination. Therefore, the enhancement of the solar cell performance with Al_2_O_3_ interfacial layer is mainly attributed to passivating the silicon surface that reduces the surface recombination and the creation of higher SBH that facilitates the transport of charge carrier effectivity.

**Fig. 7:**
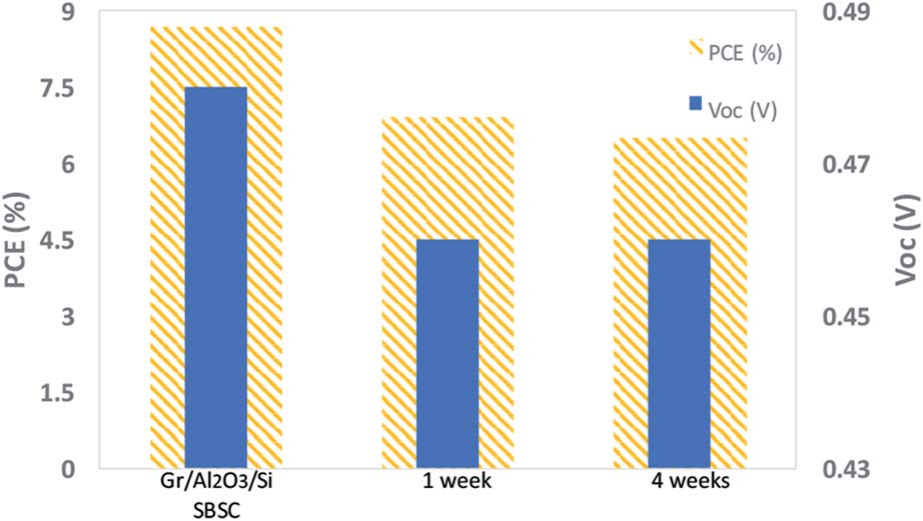
Variation of PCE and *V*_oc_ of Gr/Si with Al_2_O_3_ interfacial layer over time.

The stability of the cell was examined over four weeks as shown in Fig. 7. There was an observed degradation in the performance of the cell after one week. The drop in the efficiency and *V*_oc_ after one week is mainly attributed to the degradation effect of nitric acid dopant as observed by Cui *et al.*^13^ The nitrite anions in nitric acid acts as a p-type dopant to the graphene (*i.e.* increasing the work function of the graphene) and reduces its sheet resistance. Upon the degradation of the dopant, the graphene work function decreases and the sheet resistance increases which results in a large drop in the efficiency and *V*_oc_.

After four weeks, there was no observed major degradation in the cell performance. This highlights the benefit of using Al_2_O_3_ as a passivation layer and blocking layer since it prevents further growth of native oxide and helps the cell to maintain its performance. The slight reduction in the PCE from 6.9% in week 1 to 6.6% in week 4 is mainly attributed to the reduction in FF from 54.12% to 52.10%. The contact series resistance increased due to the use of silver paste as the top contact. The silver paste degrades more compared to thermally vacuum evaporated front contacts which mainly impact the series resistance and causes a reduction in the fill factor of the cell.^34^

Possible routes to further improve the efficiency of Gr/Si SBSC with Al_2_O_3_ layer is to investigate the effect of Al_2_O_3_ oxide and find the optimal thickness that maximize the cell efficiency. According to Song *et al.*^9^ increasing the interfacial layer thickness can further reduce the leakage current and contributes to increasing the *V*_oc_. However, with further increase in the interfacial layer beyond the optimal thickness, the tunnelling probability is reduced by the factor exp(−√*χd*) where *χ* is the average potential barrier of the oxide for hole tunnelling into the graphene and *d* is the interfacial layer thickness. Due to the reduction in the tunnelling probability, the charge carriers accumulate at the interface and results in increasing the recombination rate.

## 4. Conclusions

In conclusion, the use of Al_2_O_3_ interfacial layer to engineer the interface between the graphene and silicon is demonstrated. The existence of Al_2_O_3_ interfacial layer mainly contributed to increasing the open circuit voltage and fill factor. Al_2_O_3_ interfacial layer resulted in creating higher Schottky barrier height that is beneficial to increase the built-in potential and reduce recombination of charge carriers and leakage current. Solar cells with Al_2_O_3_ showed an improvement in the power conversion efficiency from 7.2% to 8.7% and good stability over four weeks. The results highlight the potential of using Al_2_O_3_ as interfacial layer for Gr/Si SBSC.

## Conflicts of interest

There are no conflicts to declare.

## Supplementary Material
